# Shifting dynamics: Changes in the relationship between total fertility rate and contraceptive prevalence rate in Jordan between 2012 and 2017

**DOI:** 10.12688/gatesopenres.13188.1

**Published:** 2020-10-15

**Authors:** Kristin Bietsch, Ali Arbaji, Jennifer Mason, Rebecca Rosenberg, Malak Al Ouri

**Affiliations:** 1 Avenir Health, Glastonbury, CT, 06033, USA; 2 Abt Associates, Amman, Jordan; 3 Office of Population and Reproductive Health, USAID Bureau of Global Health, Washington, DC, 20003, USA; 4 Ministry of Health, Amman, Turkey

**Keywords:** Fertility, Contraception, Jordan

## Abstract

**Background:** Between the two most recent Population and Family Health Surveys, Jordan saw a dramatic decline in the Total Fertility Rate (TFR) from 3.5 to 2.7 in 5.5 years.  Over the same period, modern contraceptive use also declined, from 61.2% to 51.8% among married women.  This decrease in both TFR and the contraceptive prevalence rate (CPR) diverges from the typical relationship seen between these two factors whereby historically as CPR increases, TFR decreases.  This paper explores this unique pattern using multiple methodologies.

**Methods:** First, we validate the survey data using nationally collected data on fertility and contraceptive distribution.  Second, we look to changes that have historically influenced changes in CPR and TFR, including changes in ideal family size and wanted fertility rates. Third, we explore proximate determinants and other influences on fertility and changes in contraception, examining the changes in the method mix and unmet need; marriage patterns, including the demographics of the married population, spousal separation, and time since last sex; postpartum insusceptibility; infecundity, both primary and secondary; and abortion, to see if any have shifted significantly enough to allow for fertility to decline with less contraceptive use.

**Results: **We find that the decline in fertility in Jordan was driven by a reduction in mistimed or unwanted pregnancies and there was a significant increase in the share of reproductive aged women who are infecund. We also concluded that the changes in fertility and contraceptive use are driven by changes in Jordanian nationals, not by the growing Syrian refugee population.

**Conclusions:** Jordan is not the only country to be experiencing a shift in the typical relationship between CPR and TFR.  Results can inform both future approaches for family planning programs and our expectations regarding what kind of change our family planning investments might buy.

## Background

In most countries, total fertility rate (TFR) and contraceptive prevalence rate (CPR) change over time in a predictable relationship wherein CPR increases while TFR declines. This pattern is consistent with both the demographic transition theory ^1^ and proximate determinants of fertility ^2^. Until recently, Jordan has followed this typical pattern. However, between the two most recent Jordan Population and Family Health Surveys (JPFHS), rather than the CPR increasing and the TFR decreasing, Jordan saw a decline in both CPR among married women (from 61.2% to 51.8%) and TFR (from 3.5 to 2.7) over the period of just 5.5 years. Was this significant shift due to data errors? If not, what changed to influence TFR and CPR in this unexpected way?

This paper aims to explore several potential factors that may have influenced this pattern change and provide further analysis of the recent past, current, and potential future trends of fertility and family planning in Jordan. We start by validating the 2017 JPFHS data for fertility and family planning by comparing them to service statistics collected by Jordan’s Department of Statistics and Ministry of Health. We then explore typical factors that may be responsible for the shift that might explain this new CPR/TFR pattern, including the ideal family size, wanted total fertility rates, unintended pregnancies and method mix.

Jordan’s unique country context as a middle-income country in the Middle East means that the specific factors contributing to its CPR and TFR pattern may not be directly comparable to other low-and middle-income countries. However, Jordan is not the only country in which we are seeing shifts to the typical relationship between TFR and CPR, and expanding our understanding of these new and unexpected patterns can inform both future approaches for family planning programs and our expectations regarding what kind of change our family planning investments might buy that will benefit other countries seeing similar changing patterns. We hope that the approaches used in this paper to analyze JPFHS and national health management and information system data and to coordinate those findings with country context and family planning programming efforts may provide discussion and learning opportunities for global family planning teams.

### Background on family planning in Jordan

Jordan’s family planning program is stewarded by the Ministry of Health (MOH) and includes long standing services in the public and private sectors which offer widespread access to contraception across the country. Approximately 49% of family planning services are supported the by the MOH, which in addition to MOH facilities, includes Royal Medical Services and public university facilities ^3^. The remaining 51% of FP services are provided by the private sector which includes private clinics, hospitals, and pharmacies, the United Nations Relief and Works Agency (UNWRA) and NGOs ^3^. There is no emergency contraceptive product available in the public and private sector and abortion is illegal except to save the life of the mother or to preserve her physical health ^4^, ^5^. To address high rates of unmet need for family planning and overcome the stagnation on contraceptive use, the MOH has supported efforts to shift social norms regarding family planning use and family size and improve the availability and quality of FP services ^6^. In addition to programmatic efforts to influence demand and use of family planning services; major recent changes in the Jordanian socio-economic environment have likely influenced fertility and contraceptive use outcomes ^7^.

Since 2011, Jordan has received hundreds of thousands of Syrian refugees - with estimates ranging from 650,000 to 1.2 million refugees over the course of six years ^7^. Of the registered Syrian refugees, an estimated 80 percent live within Jordanian towns and cities and utilize national and local services for daily life ^8^. Providing support to the refugees put additional strain on limited national resources, which resulted in challenges to fully fund and address critical health issues ^7^. The impact of the regional conflicts and refugee settlements has had a substantial economic impact on Jordanians, due to increased costs of foods, goods, and rent alongside continued high rates of unemployment and underemployment ^9^.

### Recent changes in family planning and total fertility rate

In the past, the relationship between TFR and CPR in Jordan followed the typical pattern, with TFR declining as CPR increased, as can be seen in Table 1 which shows Jordan’s TFR, CPR, modern CPR (mCPR), and traditional CPR (tCPR) from the JPFHS from 1990 to 2018. Between 1990 and 2002 Jordan’s TFR fell by almost 2 children per woman (from 5.6 to 3.7), then over the next decade (2002–2012) TFR remained fairly level, ranging from a high of 3.8 to a low of 3.5. At the same time that fertility was rapidly declining (1990–2002), total CPR increased from 40% to 56% for married women and during the 10-year stall in fertility decline, CPR increased by only 5%. Traditional methods make up a large share of the method mix in Jordan and have fluctuated in use over time. Between the 2012 and 2017–18 surveys, TFR began to decline again, by 0.8 children per woman, while at the same time CPR declined from 61% to 52% of married women. Declines of 5% each were seen in both mCPR and tCPR.

**Table 1.** Trends in fertility and contraceptive use in Jordan Population and Family Health Surveys 1990 through 2017–18.

Survey	Total fertility rate 15-49	Married women currently using any method of contraception	Married women currently using any modern method of contraception	Married women currently using any traditional or folk method
2017-18 JPFHS	2.7	51.8	37.4	14.4
2012 JPFHS	3.5	61.2	42.3	18.9
2009 JPFHS	3.8	59.3	42.0	17.2
2007 JPFHS	3.6	57.1	41.9	15.2
2002 JPFHS	3.7	55.8	41.2	14.6
1997 JPFHS	4.4	52.6	37.7	14.9
1990 JPFHS	5.6	40.0	26.9	13.1

## Methods

### Validating survey results from the 2017–18 JPFHS

We seek to validate that the changes seen in the 2017 JPFHS data are a true reflection of fertility and contraceptive use in Jordan, and not the result of data collection error. This validation is done internally, using previous JPFHS rounds, and externally using nationally collected data from the 2015 Census, birth registrations, and contraception distribution recorded by the Ministry of Health. Analysis is conducted using R version 3.6.1 ^10^.

The JPFHS is a nationally representative survey which aims to provide estimates of basic demographic and health indicators. Implemented by the Jordan Department of Statistics (DOS) with assistance from ICF through the Demographic and Health Survey (DHS) Program, the 2017–18 JPFHS is the seventh DHS to be conducted in Jordan ^3^. It is designed to be representative of the country as a whole, of urban and rural areas separately, of the three regions of Jordan, its 12 administrative governorates, and of its three national groups (Jordanians, Syrians and other nationalities) ^8^. The sampling frame is based on Jordan’s Population and Housing Census frame for 2015 and the sampling design is a stratified two-stage cluster design ^8^.

***Internal validation of the JPFHS.*** One hypothesis for the reported rapid decline in TFR is that a problem with data collection in the most recent survey resulted in an undercounting of births. This kind of error can occur if enumerators do not collect full birth histories ^11^. To see whether this type of error was present in the JPFHS, annual TFRs were constructed from the last three surveys to confirm that TFRs from the same year in multiple surveys overlap. Annual age specific fertility rates were also calculated for the 2017 survey.

To internally validate contraceptive use, we examined the contraceptive calendar collected by the JPFHS for 72 months before the survey. We constructed monthly CPR and mCPR for married women, aged 15–44, from the 2017 JPFHS contraceptive calendar. Because we only know marital status at time of the survey and date of first marriage, we excluded monthly observations from women prior to their first marriage. It is possible some women were formerly married and then remarried at some point in the calendar, but we were unable to distinguish these periods. We excluded women over 45 because we do not have women 45–49 in earlier years of the calendar and by the time of interview, they would have been above the age of 50 and excluded from the survey. We aimed to use the contraceptive calendar to see if the difference between the current use measure from the previous survey and the monthly measure from the calendar for the same time period was similar. However, our results revealed inaccuracies in the contraceptive calendar data, so it could not be used to validate contraceptive use. Due to concerns regarding the accuracy of the contraceptive calendar data, results from this analysis are not presented in the body of the text but can be found in *Extended data*, Appendix 1 ^12^.

***External validation of the JPFHS.*** To externally validate fertility rates in the JPFHS, data from the 2015 census ^13^ provided by the Civil Status and Passport Department was used to calculate TFR for Jordanian women living in Jordan. We also estimated the crude birth rates (CBR) and general fertility rate (GFR) from 2007 to 2018 using publicly available birth and population data from Jordan’s DOS ^14^. The DOS uses this data to calculate annual estimates (for the whole population and for women of reproductive age). The population data is released as end year populations. From the DOS’s annual estimates, we estimated the person-years lived annually for the entire population and women of reproductive age to use as the denominators for CBR and GFR. We assumed constant exponential growth throughout the year and estimated the growth rate using P2=P1*exp(rate*time). We then estimated the mid-year population using this growth rate. We assumed the mid-year population was equivalent to the person-years lived in the period. 

To externally validate the trend in contraceptive use between JPFHS surveys, we construct an annual estimated modern use (EMU) value using data from the Ministry of Health Contraceptive Logistic Management Information System. EMU is a measure developed by the Track20 project as part of the monitoring process supporting the FP2020 global initiative to facilitate annual estimates of modern contraceptive use based on service statistics. The tool used to generate the EMU value uses government data on contraceptive distribution, making several adjustments for long term methods distributed in years prior that may still be in use in the current year, method discontinuation, and lack of data from certain private sources (especially pharmacies and shops) ^14^. Data from 2005 to 2018 for commodities distributed to clients, commodities distributed to facilities, and family planning visits were all available for the majority of public sector and minority of private sector distribution. These variables are collected to monitor the distribution and dispensing of family planning commodities and to report to the MOH indicators related to couple years of protection (CYP) and family planning visits. Each facility receiving commodities from the MOH must report to the central MOH at the end of each month in paper format. Staff at the central level enter the data into the logistics information system. This system generates indicators including those related to stockouts. In order to use the EMU to validate the latest JPFHS mCPR estimate, we run the Family Planning Estimation Tool (FPET) with the EMU value calculated from commodities to clients data (which allows FPET to consider the service statistics data in its estimate calculations) and excluded the 2017 survey. The FPET is part of joint work by Track20, the United Nations Population Division, and UMass Amherst to estimate and project likely paths of CPR, mCPR and unmet need. It is the official tool used in tracking progress for the FP2020 Global Initiative ^15^.

### Exploring influencing factors on TFR and CPR

There are several factors that have historically been found to influence TFR and CPR. Using descriptive statistics, we explore these factors as the potential source of the change in Jordan.

***Changes in population distribution.*** One area for exploration is whether changes seen between 2012 and 2017 are driven by shifts in population distribution, notably changes in nationality. To look at this we constructed TFRs separately for Jordanians and Syrians in 2012 and 2017. Both JPFHS rounds only interviewed ever married women but TFR requires data from all women to be calculated. The JPFHS provides an all women adjustment weight for these calculations; however, subpopulation estimates require special attention ^16^. In 2017, the JPFHS provided a special all-women factor for tabulations based on nationality, allowing for TFR calculations by nationality. The 2012 survey does not provide such a weight. To compare TFRs by nationality between surveys, we used an alternative approach: because non-marital childbearing is uncommon in Jordan, we used information from the household registers to estimate the population of never married women of reproductive age. Assuming never married women have not had children, we added these women to the ever-married women sample to form an all women sample. We also made an assumption regarding age. Century month codes for women’s date of birth and date are used to construct TFR, but the household roster only provides full years of age. We assumed that on average, women are halfway through a single year age group.

***Changes in fertility intentions.*** We look at changes in wanted TFR (WTFR) and unmet need. Ideal number of children is calculated by asking women if they could go back in time before having children and choose how many children they would have, what would be the number of children they choose. WTFR is calculated in the same manner as the TFR, but only includes live births that were at or below the ideal family size of the respondent at the time of conception. Mathematically, WTFR must always be smaller than TFR, while this is not the case for ideal number of children, which can exceed TFR.

Unmet need is a measure of women who are fecund and currently trying to avoid pregnancy but are not currently using a contraceptive method, or who are pregnant or postpartum infecund and did not want to become pregnant at the time of their most recent pregnancy. The DHS algorithm for unmet need is used in this study and can be found on the DHS website ^17^.

***Proximate determinants of fertility.*** We explore determinants of fertility to understand how fertility is able to decline in Jordan given the observed decline in contraceptive use. To begin our analysis of fertility change, we calculate the proximate determinants of fertility, as described by Bongaarts ^2^. With the JPFHS data, we calculate the index of proportion married, index of contraception, and index of lactational infecundability. Due to a lack of JPFHS and national data on abortion, it was not possible to calculate the index of abortion, but theories regarding the impact of abortion on fertility are discussed in a later section.

We also conduct analysis for other factors which may limit fertility that are in line with Bongaart’s framework ^2^. Additional factors included changes in the method mix; marriage patterns, including the demographics of the married population, spousal separation, and time since last sex; postpartum insusceptibility; infecundity, both primary and secondary; and abortion. 

**Method mix** If the method mix changes so that the contraceptives used are more effective (result in fewer pregnancies from method failures), then it would be possible to experience declines in TFR with declines in CPR. We look at the method mix overtime to see if changes in method efficacy are enough to offset declines in overall method use.

**Marriage and exposure to risk of pregnancy** The JPFHS only interviews ever married women. For a greater understanding of the demographic structure of the married population, we calculate marriage by age for all women in the households interviewed by the JPFHS. Information about these women is provided by one household member who completes the household roster. Information includes age, sex, marital status, and nationality. For demographics of currently married women, we look at the women’s questionnaire to find information on time since first marriage, spousal separation, and time since last sex.

**Infecundity** To compare levels of infecundability, we look at primary sterility (the percent of ever married women aged 45–59 with no children) and the proportion of infecund/menopausal married women as categorized by the definition of unmet need (married five or more years ago, had no children in past five years, never used contraception and said "can't get pregnant" when asked whether they wanted future children). And increase in infecundity will result in fewer births and more women of reproductive age without a need for, and therefore less likely to use, contraception.

**Abortion** Both the 2012 and 2017 JPFHS record pregnancies and pregnancy outcomes as part of the contraceptive calendar. Pregnancy outcomes are recorded as births or terminations. For the most recent termination in the calendar, women were asked to identify the type of terminations (miscarriages, abortions, or stillbirths. As we do not expect the miscarriage rate to change over time, we look at women who had a pregnancy end in the calendar (either through birth or termination) to estimate if abortion increased between surveys.

## Results

### Validating survey results from the 2017–18 JPFHS

**Figure 1.**
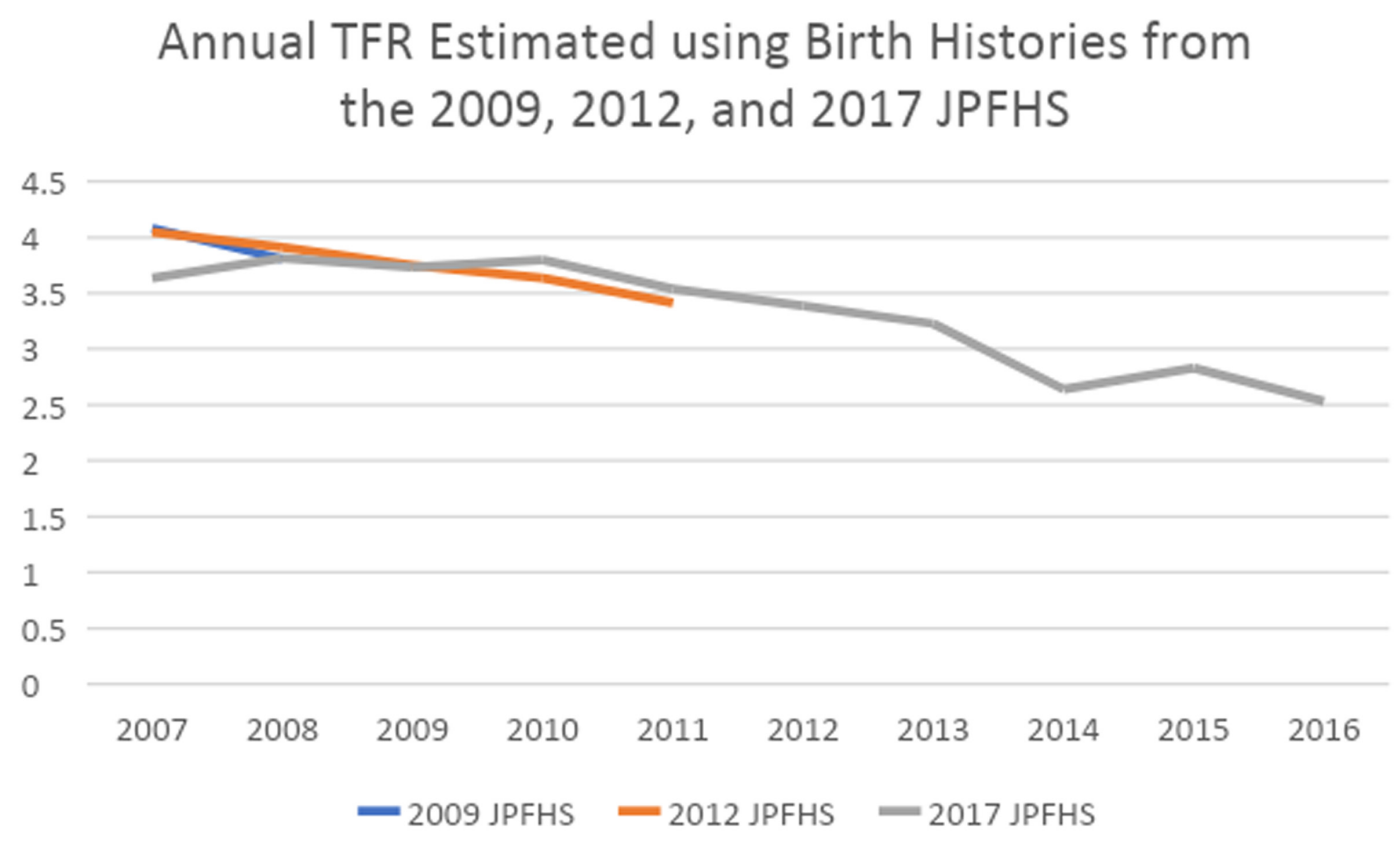
Annual total fertility rate, estimated using birth histories from the 2009, 2012 and 2017 Jordan Population and Family Health Survey.

***Internal validation of the JPFHS.*** As shown in Figure 1, we find no evidence to support the hypothesis that the reported rapid decline in TFR is due to an undercounting of births in the most recent survey. As can be seen below, except for the 2007 calculated from the 2017 survey, the TFRs are consistent. Looking at TFRs from the 2017 survey, fertility decline began around 2010 (though the older surveys show a decline from at least 2007), and a major decline in TFR happened between 2013 and 2014; there was a small bounce back in 2015, but then a decline in 2016 to the same level as 2014.

**Figure 2.**
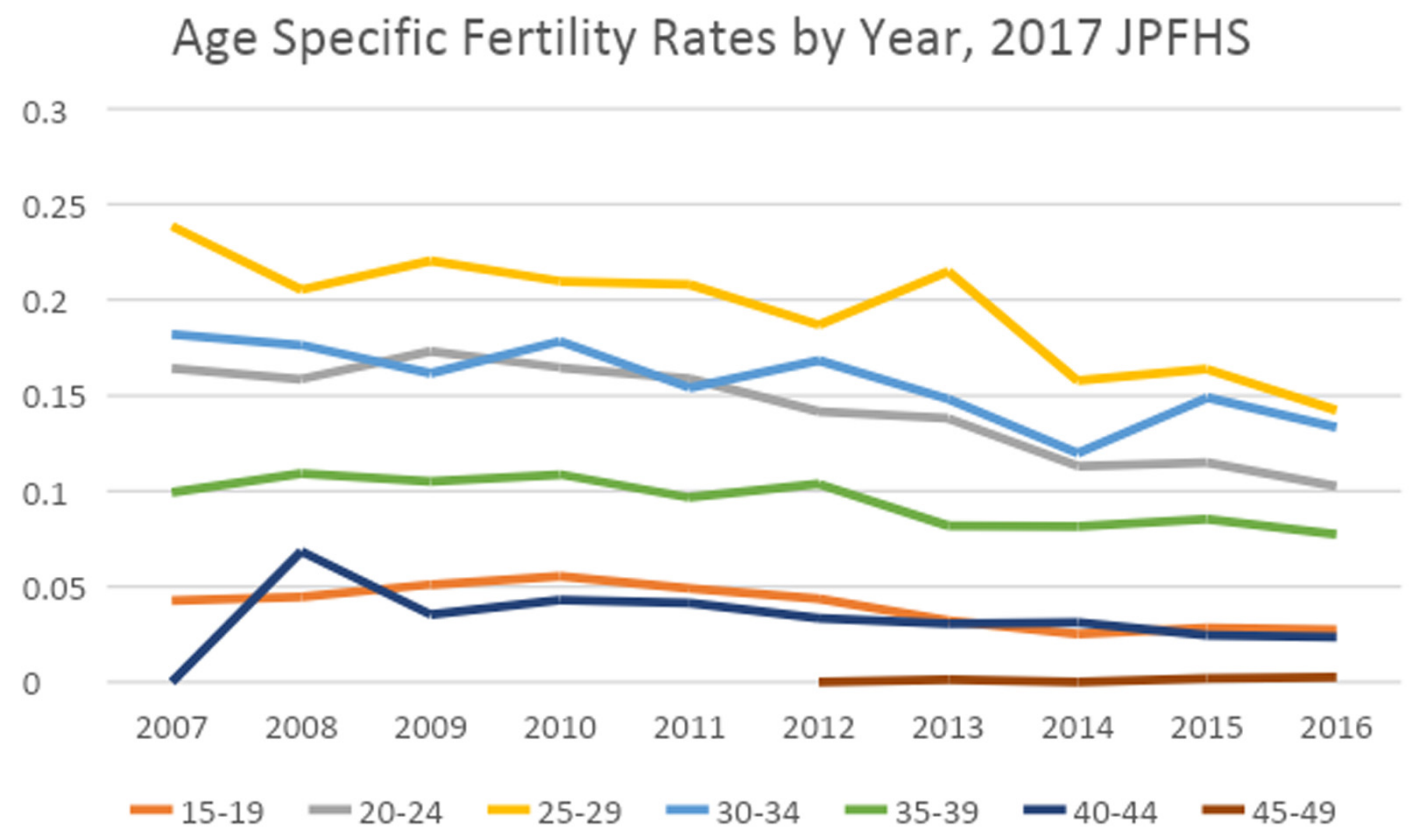
Age-specific fertility rates by year, 2017 Jordan Population and Family Health Survey.

Figure 2 shows that the drop from 2013–14 happened for the three highest fertility age groups- women ages 20–34. The drop in the three groups in 2014 is followed by one year of growth or stagnation, then a decline between 2015 and 2016 for the four age groups with the highest age specific fertility rates. There does not appear to be a data quality issue in underreporting of births in the 2017 JPFHS in comparison to earlier surveys. Fertility was falling by 2010, and may have started earlier, with a rapid decline between 2013 and 2014. 

**Figure 3.**
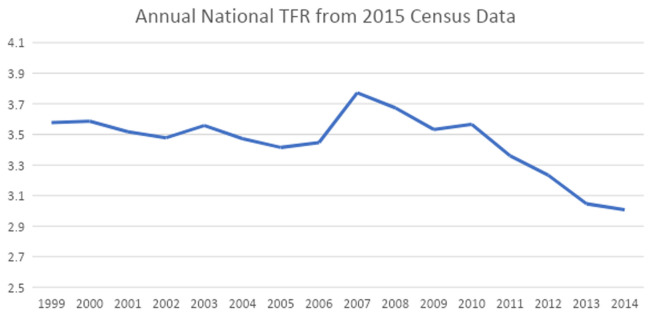
Annual national total fertility rate from 2015 Census data.

***External validation of the JPFHS.*** The national TFR for Jordanian women, which was calculated from the 2015 census, is presented in Figure 3. We can see that the TFR increased between 2006 and 2007, then started declining (as was also seen when looking at the 2009 and 2012 JPFHS), with a stall between 2009 and 2010. After 2010, it continuously declined, reaching about three children per woman three years prior to the last survey. The fertility rates calculated from census data confirm the rapid drop of TFR as seen in the JPFHS over the last five years. 

To look at more recent declines, we calculate annual estimates for CBR and GFR ( Table 2) and see continuous monotonic declines for both measures from 2007 to 2016, with the largest declines between 2012-2013. There are small increases in CBR and GFR between 2016 and 2017, though in 2018 the CBR and GFR declined to the same level as 2016. 

**Table 2.** Annual Crude Birth Rate and General Fertility Rate in Jordan, 2007–2018.

Year	Crude birth rate	General fertility rate
2007	30.8	120.7
2008	29.3	114.7
2009	28.1	110.3
2010	27.9	109.3
2011	26.1	103.9
2012	24.7	99.9
2013	22.9	92.9
2014	22.4	90.5
2015	21.6	87.4
2016	20.4	82.7
2017	21.3	86.2
2018	20.4	82.7

Annual TFRs, CBRs, and GFRs constructed from census and annual registered births show similar trends of fertility decline over the last decade as the DHS. 

**Figure 4.**
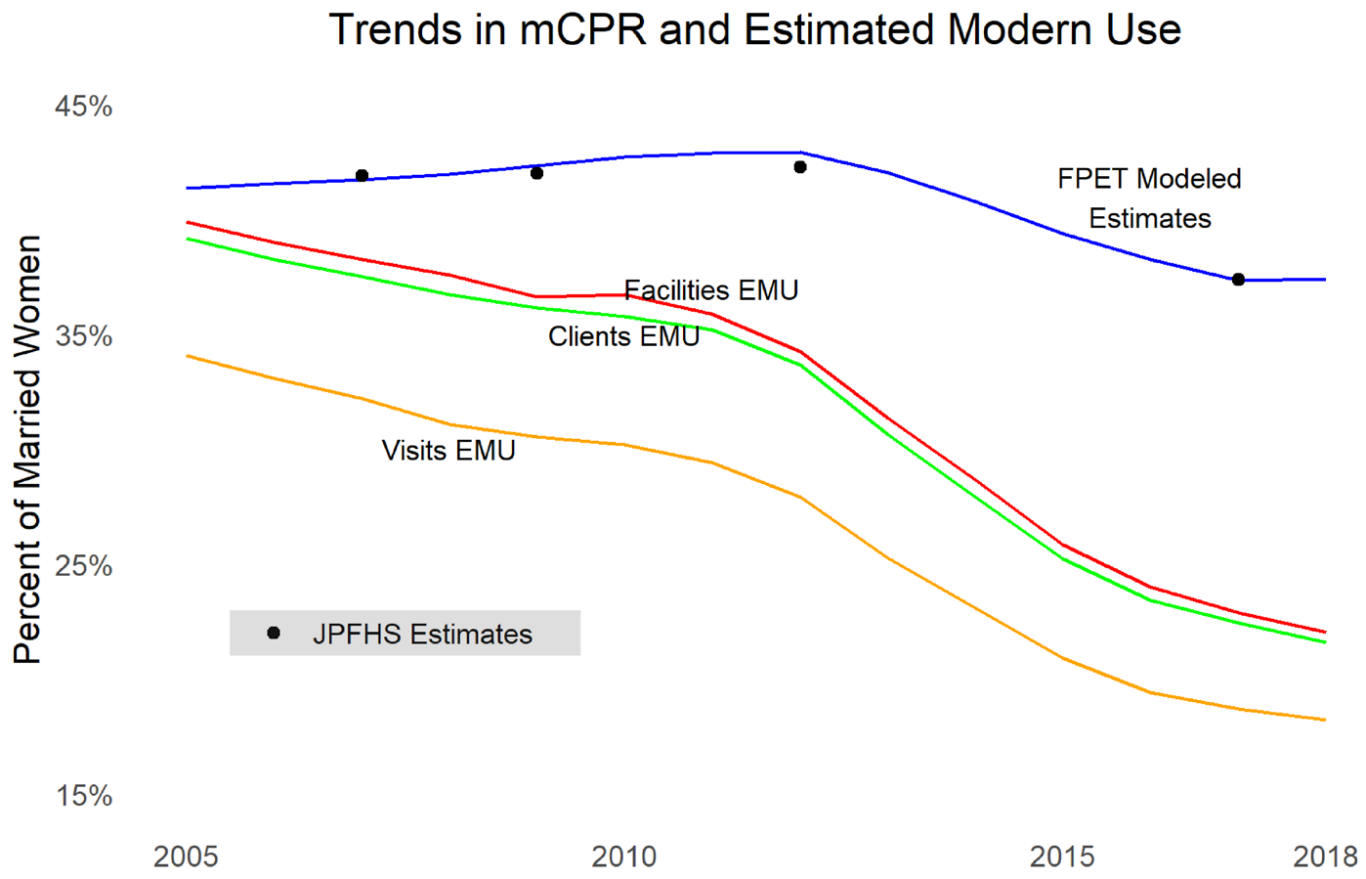
Trends in modern contraceptive prevalence rate and estimated modern use.

Turning to contraceptive use, Figure 4 shows married women mCPR from the JPFHS, modeled contraceptive trends using results from FPET using survey data (not taking into account service statistics), as well as EMU calculated for commodities to clients data, commodities to facilities data, and family planning visits data. Note that EMU does not necessarily have the same absolute level of mCPR as survey data but is examined for comparable trends. We see that all three sources of service provision data show dramatic declines, even larger than survey data (which may mean women are shifting to methods not recorded in the MOH data, such as methods from pharmacies ^3^, ^18^, ^19^ or traditional methods). The largest declines occurred after 2012. The three types of data are consistent in their trends. 

**Figure 5.**
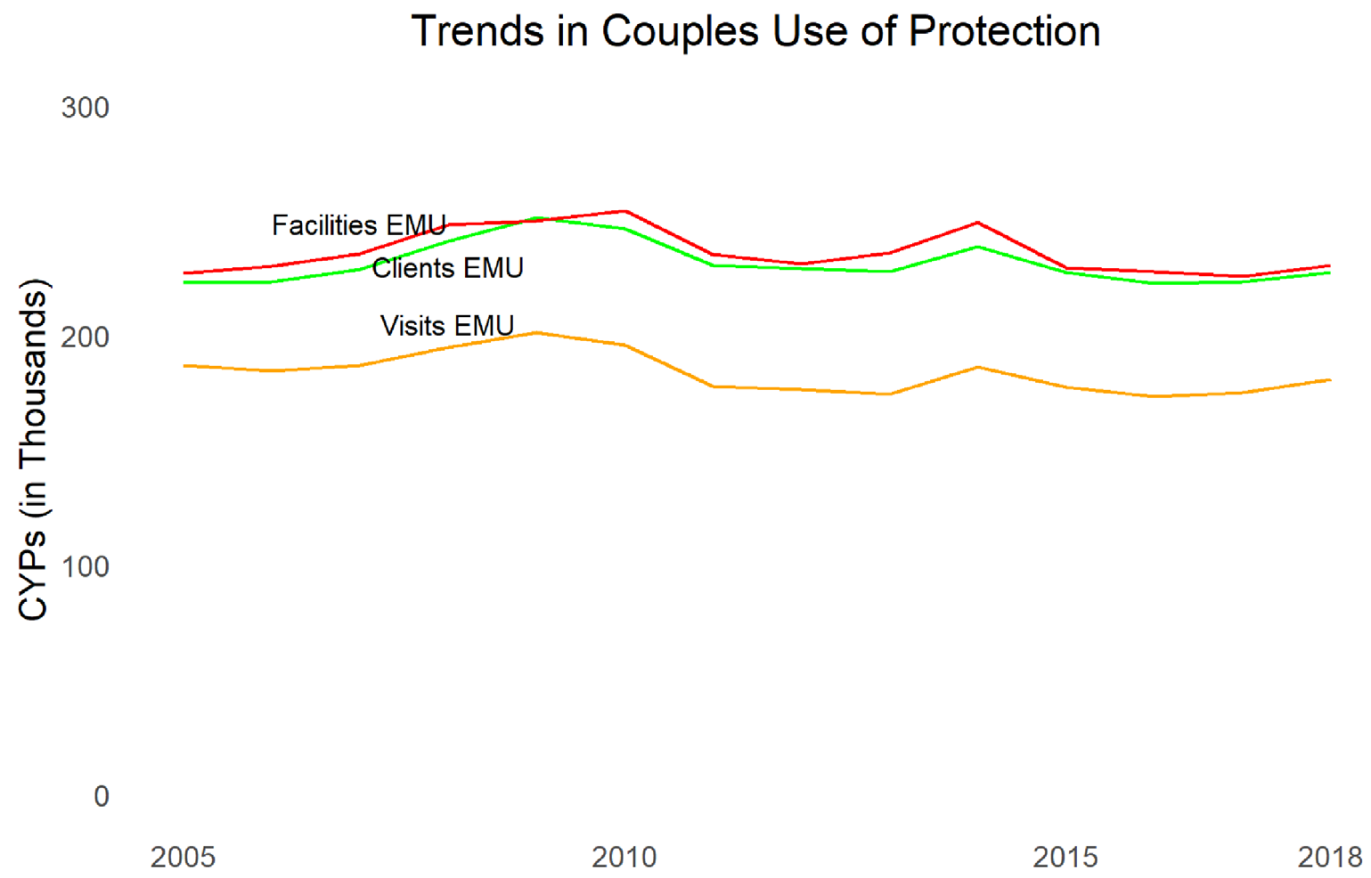
Trends in couples’ use of protection.

The EMUs show adjusted CYPs with a denominator of women of reproductive age. If we look at the CYPs without adjustment ( Figure 5), we see almost flat distribution in all three datasets. Over the same period, the number of women of reproductive age grew by 73%. A similar number of contraceptive commodities, delivered to a growing population, results in a decline of mCPR.

**Figure 6.**
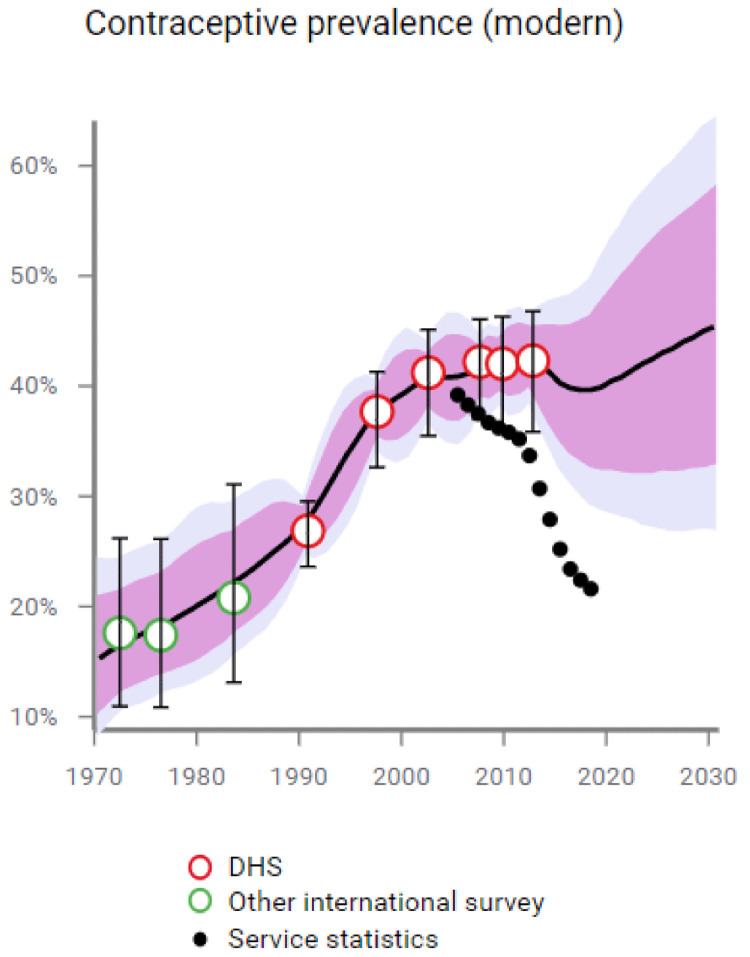
Modern contraceptive prevalence.

We ran the FPET model with EMU data, excluding the 2017 JPHFS to see if FPET would predict the decline in mCPR recorded in 2017 JPHFS. The model predicts a mCPR of 39.7, with an 80% confidence interval of 33.2% to 46.0% (shown in Figure 6), the JPFHS 2017 estimate of mCPR for married women is 37.4%, within the confidence interval and close to the mean. By including the EMU data, FPET is able to accurately predict the decline in mCPR for married women, leading us to our conclusion that service statistics data, collected continuously by the government, supports the decline in mCPR found in the latest JPFHS.

The changes seen between the 2012 and 2017 JPFHS show declines in both TFR and contraceptive use. One possible explanation of these declines are biases in survey results. Our analyses find government sources of data, including census results, birth registries, and MoH family planning service statistics mirror trends seen in the JPFHS. Since the results do not appear to be caused by errors in data collection, we turn to potential demographic explanations, which would allow TFR and CPR to decline at the same time. 

### Exploring influencing factors on TFR and CPR

To understand the declining fertility and contraceptive use, we look more closely at other factors such as women’s ability to become pregnant and use of fertility control methods that are not reported in the national service statistics.

***Changes in population distribution.*** After confirming the decline in the TFR above, we examine the declines in the TFR and changes in fertility intentions between 2012 and 2017. When possible, we look at changes by nationality, as Jordan has seen a change in its population distribution between the two surveys due to an influx in Syrian refugees. The 2012 Women Recode file from JPFHS does not include nationality. Nationality was determined here by the information provided in the Person Recode, which is matched with individuals in the Women Recode. In 2012, Jordanian nationals were 93.0% of ever married women interviewed while 2.9% were Syrian. In 2017, the Jordanian population was 86.9% of ever married women, and the share of Syrians rose to 8.6%. The remainder of the population in both surveys is categorized into Egyptian, Iraqi, other Arab Nationalities, and non-Arab nationalities. Because of their small sample sizes, we include these non-Syrian populations in total numbers, but not in breakdowns by nationality.

Table 3 shows TFRs from both the published reports and using our method of calculating TFR. When data exist in both areas it matches very closely (the largest difference is 0.1 for Syrians in 2017). Overall, there was a 0.8 child decline in TFR between 2017 and 2018. The Jordanian population also saw a 0.8 child decline in TFR, and its TFR in both surveys is only 0.1 lower than the national. Syrians saw a large decline in TFR (though it should be noted their population in 2012 was small, therefore there is much larger uncertainty in the estimates) of 1.3 children per woman. The Syrian TFR is 2 children higher than the national TFR. Both groups experienced large declines in TFR over the period, with Jordanian TFR near the national level.

**Table 3.** Total fertility rates by nationality, JPFHS 2012 and JPFHS 2017.

	Published 2012	Using never married women from household roster 2012	Published 2017	Using never married women from household roster 2017
National	3.5	3.5	2.7	2.7
Jordanian		3.4	2.6	2.6
Syrian		6.1	4.7	4.8

Table 4 shows the age-specific fertility rates (ASFRs) for Jordanians are lower than for Syrian in 2017 (constructed using the all women weight adjustment). Syrian ASFR peaks between the ages of 20–24, while the highest ASFR for Jordanians is 25–29. 

**Table 4.** Distribution of ASFRs by Nationality, JPFHS 2017.

Age Group	Jordanians	Syrians	National
15–19	17.2	133.3	26.9
20–24	103.9	247.5	109.4
25–29	153.0	216.4	156.1
30–34	136.7	188.7	137.1
35–39	89.7	85.3	87.8
40–44	24.9	53.5	26.9
45–49	1.4	7.5	1.6
TFR	2.6	4.7	2.7

***Changes in fertility intentions.*** Another possible explanation for declines in TFR is that the ideal family size is decreasing, which would motivate women to have fewer pregnancies and a lower fertility rate. If we look at ideal number of children in both 2012 and 2017, for the whole nation, Jordanians, and Syrians, the median and modal ideal number of children for currently married women is four. There is no significant change over time nationally in the mean ideal number of children (3.9 in 2012, 3.8 in 2017), which leads to the question: why would TFR experience a large decline between surveys when women’s desired number of children did not? This is especially interesting given that the ideal number of children is higher than the TFR. 

If we look at wanted TFR instead of the ideal number of children, as Table 5 shows, in 2012 there was a one-child difference between TFR and WTFR, and in 2017 this was reduced to half a child. This leads us to conclude that a major contribution to the decline in TFR between 2012 and 2017 (0.5 children out of a decline of 0.8 children per women) was a decline in births that were above individual women’s ideal number of children. In the context of women not exceeding their ideal number of children, this is a success. While the ideal number of children has not changed, the women individually exceeding their ideal number has declined, however this also implies that many women have fewer than their ideal number of children.

**Table 5.** Unintended pregnancies and mistimed or unwanted births.

	JPFHS 2012	JPFHS 2017
Wanted TFR	2.5	2.2
TFR	3.5	2.7

There is a significant decline in the percent of current pregnancies that are reported as mistimed or unwanted from the 2012 to 2017 JPFHS. In 2012, 28.8% of currently pregnant women report their birth as mistimed or unwanted, this number halved to 14.2% in 2017. There is also a decline from 29.5% to 15.5% in the percent of women who reported their last birth as mistimed or unwanted. 

The decline in the gap between WTFR and TFR, as well as the decline in the percent of pregnancies and births reported as unwanted or mistimed indicates that a large majority of the TFR decline was caused by a reduction in unwanted or mistimed fertility and took place in the absence of changes in the ideal family size. However, since there was not an increase in contraceptive use or an increase in more effective methods of contraception, the means of reducing unwanted fertility remains unexplained. Next, we turn to the desire for future births for more clues into the decline.

**Figure 7.**
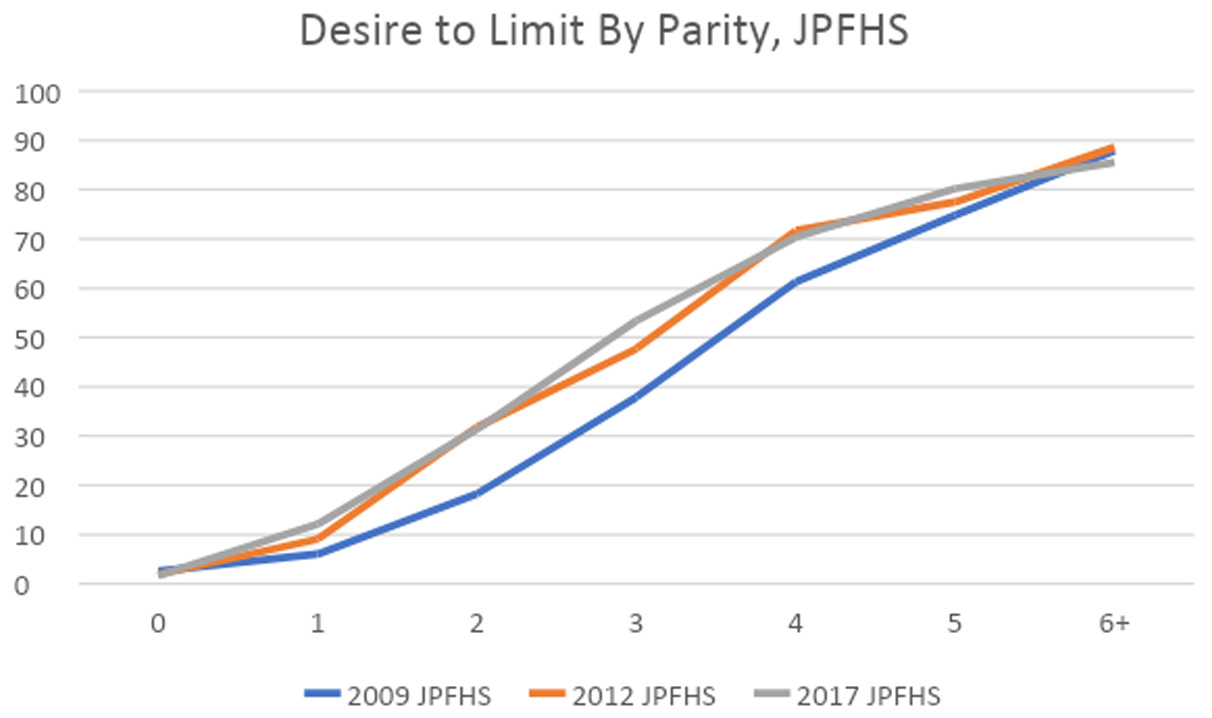
Desire to limit by parity.

**Desire for future births** Figure 7 shows the percent of currently married women who desire to limit their future births plotted against their current parity. We see that for most parities, the largest change happened between the 2009 and 2012 surveys. This finding, combined with the decline in the gap between WTFR and TFR, as seen in Table 5, suggests that desire to limit had increased by 2012, but the actual avoidance of future births was seen in the following years (the WTFR and TFR are calculated for the 3 years prior to the 2017 survey). The only parity in 2017 with a sizable increase in the desire to limit is parity 3. If more of these women do limit future births, TFR may continue to decline. 

If we look at distribution of currently married women from the 2012 and 2017 surveys, we find more uncertainty about future childbearing: in 2017, 7.1% of women are undecided if they would like to have another child, compared to 2.2% in 2012 ( Table 6). 

**Table 6.** Fertility intention of married women.

Among Currently Married Women	JPFHS 2012	JPFHS 2017
Want Another Child	41.8	36.9
Undecided	2.2	7.1
Want No More Children	50.5	47.7
Sterilized (Respondent or Partner)	2.3	1.5
Declared Infecund	3.2	6.9

The increase in the percent of married women who are undecided about having another child, coupled with a fertility rate lower than ideal family size suggests potential uncertainty about the future, which is causing women (or couples) to restrict childbearing during the time of the survey period. The decline in fertility is mainly caused by a reduction in unintended pregnancies and births, suggesting couples have more motivation to avoid these births. It is not yet known whether this is postponement in fertility, or if women will remain at the new, lower fertility rate into the future. 

**Unmet need** Unmet need for contraception increased by 2.5 percentage points from 2012 to 2017, as seen in Table 7, a statistically significant increase, although that increase was much smaller than the decrease in contraceptive use. While the government service statistics showed a steady distribution of contraception (which would lead to a decline in CPR given rising numbers of women of reproductive age, the rise in unmet need is not enough to account for this entire decline. Both Jordanians and Syrians experienced increases in unmet need between surveys, though only the Jordanian change was statistically significant. In 2012, Syrians had statistically higher unmet need than Jordanians, in 2017 the difference was no longer statistically significant. 

**Table 7.** Unmet need among married women.

Year	National	Jordanian	Syrian
JPFHS 2012	11.7	11.5	17.9
JPFHS 2017	14.2	13.6	18.6

***Proximate determinants of fertility.*** The results of our initial analysis around factors that are typically seen to influence TFR and CPR provide some insights into movement of one factor, but do not fully explain why in Jordan both TFR and CPR have declined together. This leads us to explore possible explanations related to proximate determinants of fertility, the results of which are presented below.

The index of proportion married, index of contraception, and index of lactational infecundability are presented in Table 8. The data used to calculate the indices are available in *Extended data*, Appendix 3 ^12^.

**Table 8.** Proximate determinants for fertility.

Survey Year	Index of Marriage (Cm)	Index of Contraception (Cc)	Index of postpartum infecundity (Ci)	Total Fecundity	Cm*Cc*Ci	Predicted Fertility Rate (No Abortion)	Observed fertility
JPFHS 2012	0.479	0.482	0.926	15.3	0.214	3.3	3.5
JPFHS 2017	0.472	0.550	0.926	15.3	0.241	3.7	2.7

Given a Total Fecundity of 15.3 (the average total fecundity used by Bongaarts ^2^), we predict a fertility rate of 3.3 in 2012, and 3.7 in 2017–18, with the main driver of change the increase in the index of contraception. There is a slight decrease in the index of marriage and no change in the index of postpartum infecundity. Given that the predicted fertility rate in 2012 is higher than the observed fertility rate, it is likely the total fecundity is higher than 15.3 in Jordan. In 2017, a predicted fertility rate one child per woman higher than the observed fertility rate signifies that the three fertility inhibiting factors included in this analysis of the proximate determinants do not fully capture all fertility limiting factors. If the only other limiting factor of fertility in 2017 was abortion, the total abortion rate would need to be 1.6 abortions per woman (Calculated using the index of induced abortion necessary for TFR=Tf*Cc*Ci*Cm*Ca, with Ca=TFR/[TFR+[0.4*[1 + % of women using contraception]* TAR]]), however, there are other factors which may lower fertility.

**Temporal measurement effects** One possible explanation for declines in TFR and CPR simultaneously is a temporal measurement issue. The fertility rate is measured over the 3 years prior to the survey, while contraceptive use is a current status measure from the time of the survey. Therefore, theoretically, contraceptive use could have been high for several years before the survey, leading to declines in the number of births, and contraceptive use might have fallen immediately before the survey. However, given the steady declines in the EMU calculated from government provided service statistics data, contraceptive use has been steadily declining for some time in Jordan. Nationally, CPR declined by 9.3 percentage points between 2012 and 2017. For native Jordanians, the decline was 8.5% (statistically significant), and 11.0% for Syrians (not statistically significant). 

**Figure 8.**
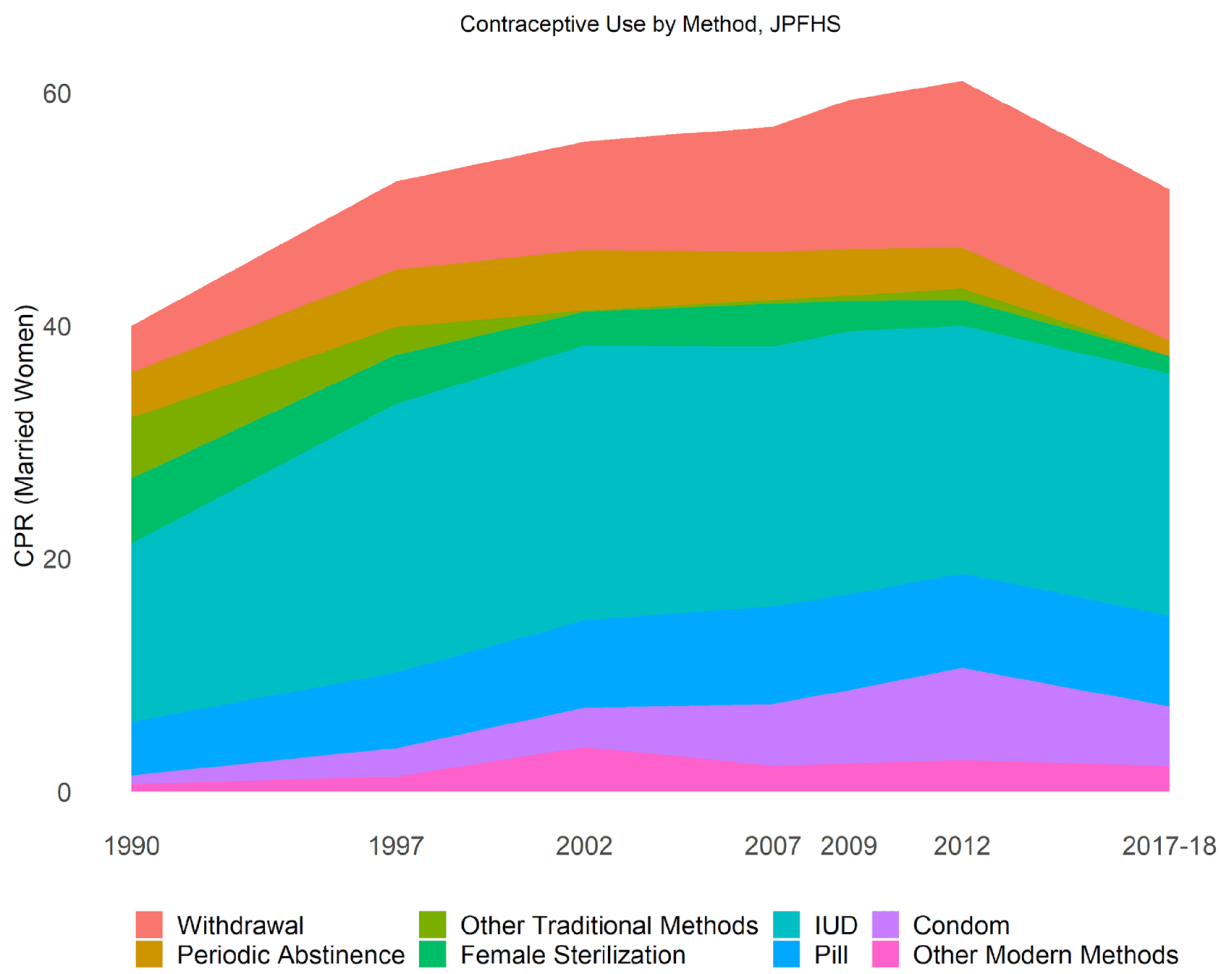
Contraceptive use by method.

**Method Mix** While CPR declined by 9.3 percentage points between 2012 and 2017 (as seen in Figure 8), only three individual methods changed by more than a percentage point each. Male condom use declined from 7.5% to 4.8% among married women (statistically significant), while periodic abstinence declined from 3.4% to 1.2% (statistically significant), and withdrawal from 13.6% to 12.1% (marginally significant). One would suspect that given the declines were concentrated in less effective methods, the average contraceptive effectiveness (which accounts for the method mix and failure rates) would increase, but we find only a slight increase in effectiveness from 90% to 91%, not enough to offset the 10-percentage point decline in CPR. 

We also looked at use of emergency contraception (EC) in Jordan. If women do not consider EC a method they “currently use,” it may be underreported in the 2017 JPFHS, and if women are switching to EC from other methods, this underreporting may help to explain why we see a decline in CPR accompanied by a decline in TFR.

Only one woman reported being a current EC user at the time of the survey (and she also reported being a pill user). Only four instances of EC use were reported in the contraceptive calendar- all by one woman in four continuous months, a potential data error. Reports of low EC use is not surprising, given that no emergency contraceptive brands are registered in Jordan ^20^. However, knowledge of EC has risen from 15.3% of married women in 2012 to 27.3% in 2017, an increase which is statistically significant. 22.5% of married men had knowledge of EC in 2017 (men were not interviewed in 2012). One hypothesis for EC knowledge being so high without a dedicated product in country is the off-label use of hormonal contraceptive pills. Though pharmacists in Jordan are not found to be well informed on EC ^4^ women may obtain guidance from providers on using the Yuzpe method ^21^and/or use information from the internet to help them use oral contraceptive pills for EC. There is no national tracking of off label use of oral contraception as an emergency contraceptive method and women may underreport in the JPFHS by reporting emergency contraceptive use as oral contraceptive pill use.

**Marriage and exposure to risk of pregnancy** Overall, 56.0% of women of reproductive age in the households interviewed by JPFHS were currently married in the 2017–18 survey, compared to 54.2% in 2012, though this increase is not statistically significant. The mean age of married women rose from 34.4 to 34.7, the largest change by age group was 45–49-year-old women, who increased as a share of married women from 12.8 to 15.8% (a statistically significant increase). At the same time, Table 9 shows that more women were likely to have been married for under two years in 2017 compared to 2012, 8.9% compared to 6.9% (statistically significant), while the percent who had been married for 2–4 years declined from 13.0% in 2012 to 11.7% in 2017–18 (not statistically significant). This postponement in marriage and change in marital distribution may explain some of the reasons for the drop in current contraceptive use. The higher proportion of women of reproductive age that were recently married could impact the contraceptive rate, because they may want to have their first child soon and may not have been using contraceptives during the period of the survey (less than 1% of nulliparous women reported using modern contraception in either the 2012 or 2017 JPFHS). Also, these women would not have been married during a large part of the 3-year windows used to construct total fertility rates. 

**Table 9.** Time since first marriage among currently married women.

Time since first marriage	2012 JPFHS	2017 JPFHS
Under 2 Years	6.88	8.85
2–4 Years	12.95	11.65
5–9 Years	20.63	19.78
10–14 Years	17.25	17.19
Over 15 Years	42.30	42.52

**Figure 9.**
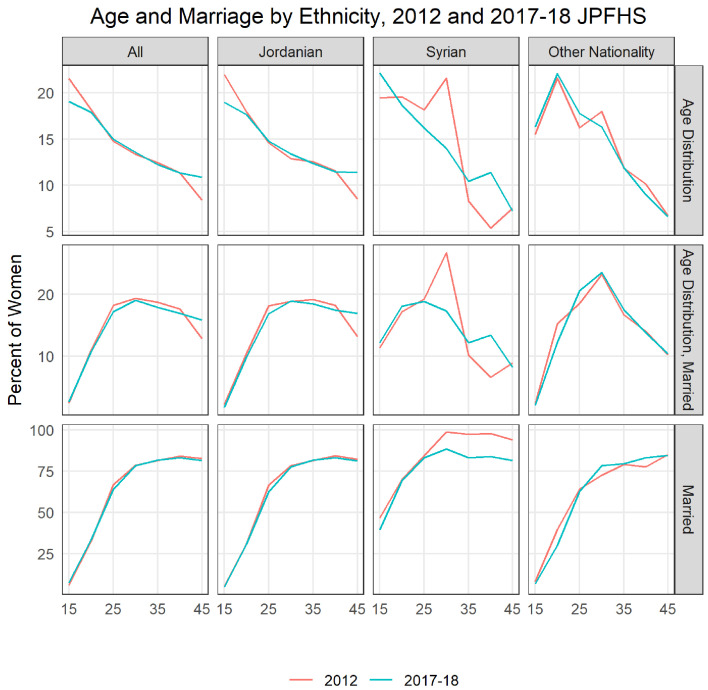
Age and marriage by ethnicity, 2012 and 2017–18 JPFHS.

Figure 9 illustrates the changing population structure of women of reproductive age by age and ethnicity. The top left corner shows the age distribution of all women of reproductive age in the country, we see that there are fewer younger women and more older women in Jordan in 2017 compared to 2012. This change is mainly driven by change among native Jordanians. The Syrian population, especially in 2017, is skewed younger than the population overall. Looking at the percent married by age and ethnicity, we see very little changes for Jordanian women and non-Jordanian, non-Syrian women. Syrian women among the younger ages are much more likely to be married than other women. Overall, 39% of 15–19-year-old Syrians were married in 2017, compared to 4.9% of Jordanians and 6.9% of other nationalities. Between the two surveys, there was over a doubling of Syrian women as a share of married women, from 3.0% to 7.8%. The overall increase in the population of Syrians led to an increase in the percent married among 15–19-year-old women to grow from 6.2% in 2012 to 7.6% in 2017–18, though overall the share of the married population who were aged 15–19 only increased from 2.5% to 2.6% (neither is a statistically significant change). 

If more married women are not living with their husbands this could explain both a decrease in contraceptive use (as they are less likely to be at risk of pregnancy) and decrease in fertility. Table 10 shows that in 2012, 2.9% of currently married women were not living with their husband, in 2017 the percent rose to 4.5%, a 50% increase (and statistically significant), though still a small proportion of the population. Syrian women were more likely to not live with their husbands than Jordanians in both surveys. Changes by nationality were not statistically different between the two surveys. 

**Table 10.** Percent of married women living with husband.

Year	National	Jordanian	Syrian
2012 JPFHS	97.1	97.5	86.9
2017 JPFHS	95.5	96.8	90.9

Decreased coital frequency could also lower both CPR and TFR; the JPFHS does not measure coital frequency but does measure time since last sexual experience. Table 11 shows that in 2012, 89.2% of married women had sex in the last 4 weeks, this share increased to 90.6% in 2017, a statistically insignificant change. Both Jordanians and Syrians experienced statistically significant increases in the percent of married women having sex in the last 4 weeks. 

**Table 11.** Percent of married women who had sex in last 4 weeks.

Year	National	Jordanian	Syrian
2012 JPFHS	89.2	90.1	74.4
2017 JPFHS	90.6	91.9	86.8

Overall, there has been a slight increase in spousal separation and no change in recent sexual activity among married women. In both surveys, women who live with their husbands are statistically more likely to use contraception, therefore, a decline in cohabitation among married women may lead to a decline in contraceptive use (and an increase in unmet need), though this would only explain a small portion of the overall decline. The increase in the share of newly married women could also decrease contraceptive use since contraceptive use between marriage and first birth is uncommon. 

**Figure 10.**
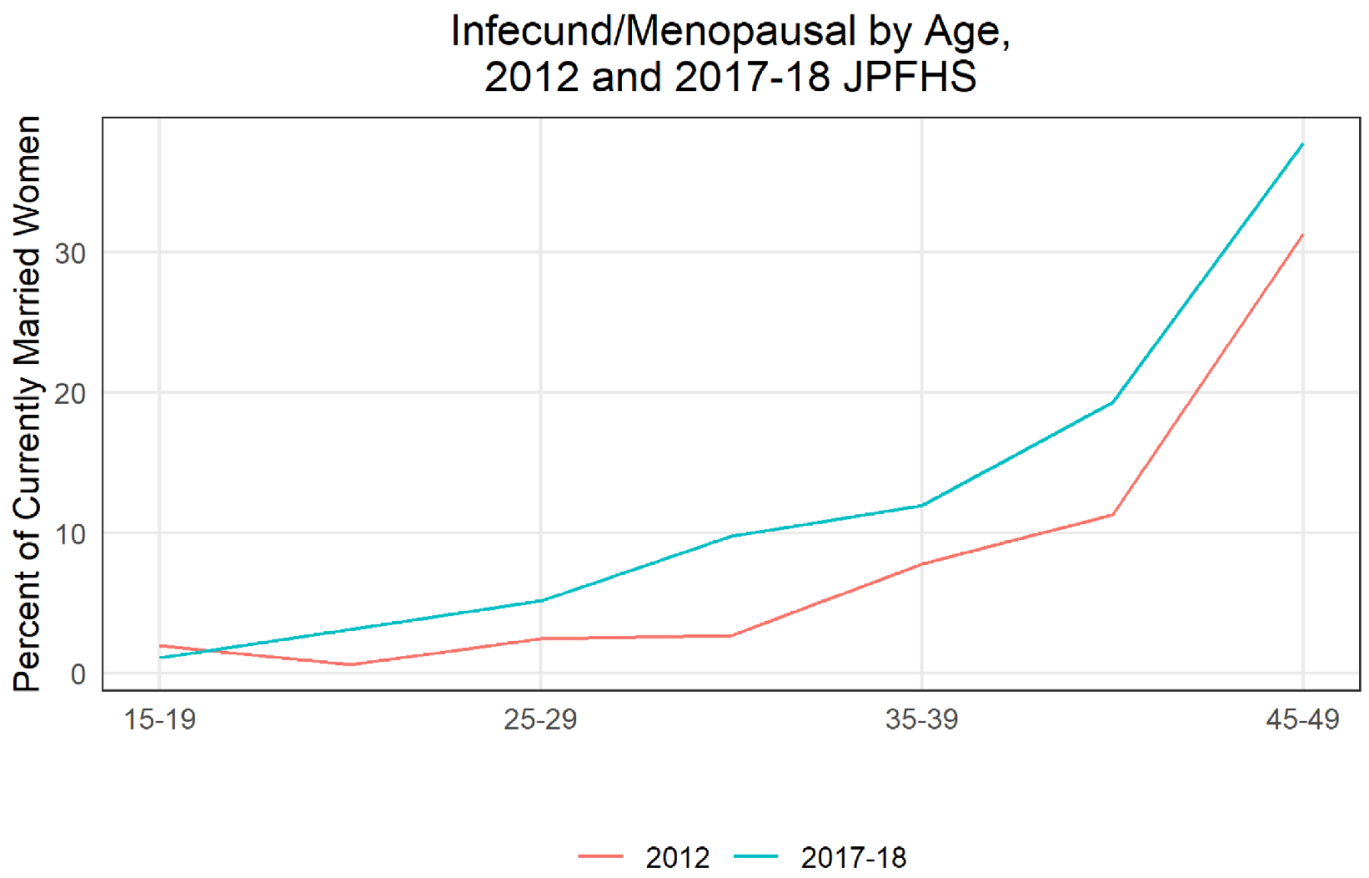
Infecund/menopausal by age, 2012 and 2017–18 JPFHS.

**Infecundity** Primary sterility declined slightly between 2012 and 2017, from 7.4% for ever married women to 6.2% (a non-statistically significant change). Secondary sterility, or the percent of women defined as infecund, rose from 8.7% in 2012 to 14.5% in 2017 (a statistically significant increase). Figure 10 shows infecundity rose in all but the youngest age group. The largest increases are seen among 40 to 44-year-old women (increase in 8.0 percentage points and statistically significant) and 30-34-year-old women (7.1 percentage points and statistically significant). The higher share of women at older ages in the population, as discussed above, increased the population level infecundity as well. Some sections of the definition of infecund are self-reported, but many others are calculated using past contraceptive use and reproductive occurrences - a large increase in the percent of women who are infecund may account for a sizeable share in the decrease of CPR (especially considering the biggest declines were in coitus dependent methods such as condoms and withdrawal) as well as declines in fertility rates. Women who are infecund may choose to not use contraception because of their lower risk of conception. Note that by definition women who are using contraception cannot be defined as infecund based on the unmet need algorithm. Therefore, it is possible that some women who are infecund or menopausal are not reported as such because they do not know their status and are currently using contraception, and the decline in contraceptive use could have unmasked a larger share of the infecund population. Public health practitioners in Jordan have identified the high prevalence of smoking, both cigarettes and traditional “shisha” pipes ^22^ (12%) and overweight/obesity ^23^ (54%) as possible reasons for increases in delayed fertility or infecundity.

**Abortion** Table 12 shows that there was a large increase in the percent of currently married women who reported no pregnancy outcome in the calendar (either were not pregnant in the calendar or were only pregnant at the end of the calendar/time of interview). The percent of women whose most recent outcome was a birth declined, as did the percent that had a miscarriage or stillbirth, only abortion slightly rose. The large decline in fertility makes it difficult to compare changes between other outcomes. 

**Table 12.** Pregnancy outcomes from calendar data.

Of Currently Married Women: Most Recent Pregnancy Event Outcome	2012 JPFHS	2017 JPFHS
No pregnancy outcome in calendar	31.1	42.8
Birth	59.5	51.0
Miscarriage	8.3	4.9
Abortion	0.8	1.1
Stillbirth	0.4	0.2

Table 13 shows the distribution of pregnancy outcomes (women with no pregnancy outcomes in the calendar are removed) and finds that the percent of pregnancies ending in abortion is small, only 1.2% in 2012, and 2.0% in 2017, a statistically significant increase. The increase in pregnancies ending in abortion may signal an overall increase in the trend, however the level is notably low. Abortion is only legal in Jordan to save the life of a woman or preserve physical health ^24^. Globally, abortion is under-reported, especially in countries with restrictive policies or where it is socially stigmatized ^25^. However, given an estimated 22% of pregnancies end in abortion in Western Asia ^26^, it is very likely that abortions are more than 2% of all pregnancies and underreported in the calendar. Additionally, the large decline in miscarriages, from 12.0% in 2012 to 8.5% in 2017 is noteworthy, especially without significant public health interventions designed specifically to reduce miscarriage. While the level is close to the global average of 10% of recognized pregnancies to end in miscarriage ^27^, there does not appear to be an obvious reason for such a large decline in the trend. Consanguinity has declined in Jordan over time, though evidence of consanguinities effect of pregnancy loss is mixed ^28– 31^. Both the low reported levels of abortions and decline in miscarriage signal inconsistencies in calendar data. Further inconsistencies in the calendar, regarding contraceptive use over time are shown in *Extended data*, Appendix 1 ^12^. While abortion may be allowing for a simultaneous decline in CPR and TFR, we are unable to substantiate this hypothesis with the available data. 

**Table 13.** Pregnancy outcomes from calendar data of those who had a pregnancy in the calendar.

Of Currently Married Women with Pregnancy Outcome in Calendar: Most Recent Pregnancy Event Outcome	2012 JPFHS	2017 JPFHS
Birth	86.3	89.2
Miscarriage	12.0	8.5
Abortion	1.2	2.0
Stillbirth	0.6	0.4

## Discussion

The changes between 2012 and 2017 JPFHS provide national family planning policy makers and program managers with a conundrum - how can the TFR decline when contraceptive use also declines? What do such changes mean to the national FP program? The analysis in this paper shows that there is not a simple answer to this question and that many factors beyond the traditional elements of family planning programming influence the outcomes. When examining the data by nationality, changes in the native Jordanian population mirror changes in the national numbers, even with the changes in population composition, which indicates that the influx of Syrian and other refugees in recent years is not a significant contributing factor to this new pattern.

Several fertility inhibiting changes could have allowed fertility and contraceptive use to decline simultaneously. Primarily, the increase in infecundity, seen across most age groups, could lower both fertility rates and contraceptive use rates. Given that declines in contraception were mostly driven by declines in coitus dependent methods adds further weight to the knowledge of subfecundity among the population. While there are issues with direct measurement of both emergency contraceptive use and abortion, the data signals that there may be changes in use of both that could help reduce fertility, but not increase the CPR (especially if women do not consider EC a method they are “currently” using). For EC, the increase in knowledge without the availability of a branded EC on the market suggests there may be use off label use of oral contraceptives. There appear to be inconsistencies throughout the contraceptive calendar, but we do see a significant, though small, increase in the percent of pregnancies ending in abortions. 

The rise in share of recently married women of reproductive age (intending to get pregnant soon), an increase in proportion of women of reproductive age in the 45–49 age group (increasing the overall rate of infecundity), and a general increase in infecundity across all age groups results in a decreased need for contraception. Despite a decline in the overall need for contraception, unmet need for contraception among women who were not infecund or trying to become pregnant increased substantially within a short period of time, meaning that a significant group of women who did not want to become pregnant were not using contraception. However, the increase in unmet need was not as large as the decline in CPR.

Possibly due to a challenging economic climate and potential shifts in social norms, increasing numbers of Jordanian women are considering having no children at all, or are considering having no more children or uncertain about their plans to have additional children, yet there has been an overall decline in the use of modern and traditional contraceptive methods. This is countered by the fact that the desired number of children is still approximately four and has not changed since 2012. This information presents a picture of a complex economic and socio-cultural environment that is impacting women’s choices and practices regarding fertility and contraceptive use, suggesting that women (and couples) are having fewer children than they would ideally want and those who have their ideal number of children are less likely to exceed this number.

## Conclusion and recommendations for programming

Continued monitoring of family planning services, contraceptive uptake, and rates of infecundity are needed to determine whether the 2017 JPFHS outcomes are short term, or if they represent a trend that will last for several years. To help ensure that Jordanian women can achieve their desired fertility and optimal family size, programmatic interventions to both strengthen family planning services and demand creation along with further research and attention on women’s infecundity are needed. 

Although Jordan has been able to reduce its TFR without commensurate increases in contraceptive use; it is not clear that those declines can be sustained without increases in FP use over time. To help women achieve their fertility intentions, national efforts to improve use of voluntary family planning should be strengthened. Part of those efforts should include the application of a life cycle approach to Jordanian women’s reproductive needs and services, including the flexibility to support women’s changing family planning needs from the time they marry and desire to have children to later years when they may wish to limit fertility. Also critical in this programming is the analysis and planning for women coming into and out of infecundity - whether due to postpartum amenorrhea or subfecundity that may be associated with health or environmental factors. Further analysis of the factors driving the high rates of infecundity found in the survey should be conducted and clinical support provided to women experiencing infertility so that they can achieve their desired fertility.

In addition to continuing national efforts to improve the quality of family planning services, the expansion of the contraceptive method mix may help to attract new or discontinued family planning users ^32^. The introduction of a hormonal intrauterine device in the public sector and emergency contraceptive for both public and private sector may fill gaps in the existing method mix and efforts to reposition underutilized contraceptive methods such as injectables, implants, and sterilization, may also help to increase women’s use of contraception. Given the historically high use of withdrawal in Jordan, it may be useful for family planning programmers to conduct research on improving efficacy and counseling to enable couples to increase effectiveness of their chosen method. With the high availability of cell phones and internet access in Jordan family planning programmers should consider providing counseling to women on the use of branded internet apps to support fertility awareness methods such as the standard days and two-day methods. 

To dispel myths and misconceptions and to improve support for family planning use, social and behavior change interventions should be developed for segmented groups of potential contraceptive users and their influencers. These efforts can utilize women’s changing desires regarding fertility to influence perceptions and use of FP for the long term, which may help drive a sustained change in the TFR.

To spur the growth of contraceptive use, the MOH should focus on improving the quality of family planning services along the continuum of care. Choice of contraceptive methods, information given to clients, technical competence, interpersonal relations, follow-up-continuity mechanisms, and appropriate constellation of services are the main determinants of quality family planning services ^33^. The quality components of family planning services are expected to impact not only the choice of appropriate method but also the future behavior of the user in terms of continuation, discontinuation, or switching. The improved quality of family planning services at service delivery points (including reducing provider bias, ensuring adequate method mix with special focus on long acting methods, and elimination of stock-outs), coupled with ceasing missed opportunities such as raising awareness at the community level, are core programmatic strategies to increase use of modern contraceptives in Jordan.

## Data availability

### Underlying data

Jordan’s Demographic and Health Surveys are available in the MEASURE DHS repository ( http://www.measuredhs.com). Access to the dataset requires registration and is granted to those that wish to use the data for legitimate research purposes. A guide for how to apply for dataset access is available at: https://dhsprogram.com/data/Access-Instructions.cfm.

Jordan’s annual estimates of population and birth are available for the Jordanian Department of Statistics at http://dosweb.dos.gov.jo/population/.

Jordanian census data used to calculate Total Fertility Rates and Ministry of Health data used to calculate Estimated Modern Use were directly provided to the authors and are not openly available. If individuals would like to enquire about accessing this data, please write to Malak Al Ouri at alouri_malak@hotmail.com.

### Extended data

Zenodo: kristinbietsch/JordanFertilityFP: Release for Gates 092920. https://doi.org/10.5281/zenodo.4058480 ^33^.

File ‘Jordan TFR CPR Extended Data.docx’ contains the following extended data:

- Appendix 1: Analysis of contraceptive calendar data.
- Appendix 2: Algorithm for calculating Unmet Need.
- Appendix 3: Proximate Determinants of Fertility Data.

Extended data are available under the terms of the Creative Commons Attribution 4.0 International license (CC-BY 4.0).

## Software availability

**R code for all analysis is available at:** https://github.com/kristinbietsch/JordanFertilityFP/releases/tag/V2.4.

**Archived source code at time of publication:** https://doi.org/10.5281/zenodo.4058480 ^12^.

**License:** MIT License.

## Notes

^1^ Kirk, D. 1996. “Demographic transition theory.” *Population Studies 50* (3) : 361–387. https://doi.org/10.1080/0032472031000149536.

^2^ Bongaarts, J. 1978. “A framework for analyzing the proximate determinants of fertility.” *Population Development and Review* 4 (1):105–132. https://doi.org/10.2307/1972149. 

^3^ Department of Statistics (DOS) and ICF. 2019. *Jordan Population and Family Health Survey 2017–2018.* Amman, Jordan, and Rockville, Maryland, USA: DOS and ICF. https://www.dhsprogram.com/pubs/pdf/FR346/FR346.pdf.

^4^ El Mowafi, I. & A. Foster. 2020. “Emergency contraception in Jordan: Assessing retail pharmacists’ awareness, opinions, and perceptions of need.” *Contraception* 101 (4): 261–265. https://doi.org/10.1016/j.contraception.2019.10.002.

^5^ Singh, S, L. Remez, G. Sedgh, L. Kwok & T. Onda. 2017. “Abortion worldwide 2017: uneven progress and unequal access. https://www.guttmacher.org/sites/default/files/report_pdf/abortion-worldwide-2017.pdf.

^6^ Institute for Reproductive Health. 2016. *Final Report: Jordan Family Planning Assessment.* FACT Project. Washington, D.C.: Institute for Reproductive Health, Georgetown University. http://irh.org/wp-content/uploads/2016/05/USAID_FP_assessment_2016_IRH_FINAL_REPORT.pdf.

^7^ Alshoubaki, W. & M. Harris. 2018. “The impact of Syrian refugees on Jordan: a framework for analysis.” *Journal of International Studies* 11 (2): 154–179. https://doi.org/10.14254/2071-8330.2018/11-2/11.

^8^ World Food Program USA. 2019. *10 facts about the Syrian refugee crisis in Jordan.* World Food Program USA. https://www.wfpusa.org/articles/10-facts-about-the-syrian-refugee-crisis-in-jordan/#.

^9^ World Bank. 2019. *Jordan*. http://pubdocs.worldbank.org/en/837261553672494334/jordan-MEU-April-2019-Eng.pdf.

^10^ RStudio Team (2020). RStudio: Integrated Development for R. RStudio, PBC, Boston, MA URL http://www.rstudio.com/

^11^ Schoumaker, B. 2014. *Quality and consistency of DHS fertility estimates, 1990 to 2012.* DHS Methodological Reports No. 12. Rockville, Maryland, USA : ICF International. https://dhsprogram.com/publications/publication-mr12-methodological-reports.cfm.

^12^ Kristin Bietsch. (2020, September 29). kristinbietsch/JordanFertilityFP: Release for Gates 092920 (Version V2.4). Zenodo. http://doi.org/10.5281/zenodo.4058480

^13^ Department of Statistics (DOS). n.d. *Population and housing 2015.* http://dosweb.dos.gov.jo/censuses/population_housing/census2015/

^14^ Department of Statistics (DOS). n.d. *Population.* http://dosweb.dos.gov.jo/population/

^15^ New, JR & L. Alkema. 2015. *Family planning estimation tool (FPET).* http://fpet.track20.org/.

^16^ DHS Program. n.d. “Guide to DHS Statistics DHS-7.” DHS Program. https://dhsprogram.com/Data/Guide-to-DHS-Statistics/Analyzing_DHS_Data.htm.

^17^ https://www.dhsprogram.com/topics/Unmet-Need.cfm#:~:text=What%20is%20unmet%20need%3F,and%20has%20changed%20over%20time.

^18^ Department of Statistics and ICF. 2013. *Jordan Population and Family Health Survey 2012.* Calverton, Maryland, USA: Department of Statistics and ICF International.

^19^ The percent of modern method users who received their method from a pharmacy increased from 7.4% in 2012 to 15.1% in 2017.

^20^ International Consortium for Emergency Contraception. *EC status and availability: Jordan.* https://www.cecinfo.org/country-by-country-information/status-availability-database/countries/jordan/.

^21^ Yuzpe, A & W. J. Lancee. 1977. “Ethinylestradiol and dl-norgestrel as a postcoital contraceptive.” *Fertility and Sterility* 28 (9): 932–936. https://doi.org/10.1016/S0015-0282(16)42793-7.

^22^ Reproductive Facts. *Smoking and infertility.* Birmingham, Alabama, USA: American Society of Reproductive Medicine. https://www.reproductivefacts.org/globalassets/rf/news-and-publications/bookletsfact-sheets/english-fact-sheets-and-info-booklets/smoking_and_infertility_factsheet.pdf.

^23^ Dag, Z.O. & B. Dilbaz. 2015. “Impact of obesity on infertility in women.” *Turkish-German Gynecological Association* 16 (2): 111–117. https://doi.org/10.5152/jtgga.2015.15232.

^24^ United Nations, Department of Economic and Social Affairs, Population Division. 2014. *Abortion policies and reproductive health around the world.* New York, New York, USA: United Nations. https://www.un.org/en/development/desa/population/publications/pdf/policy/AbortionPoliciesReproductiveHealth.pdf.

^25^ Moseson, H., M. Massaquoi, C. Dehlendorf, L. Bawo, B. Dahn, Y. Zoila, E. Vittinghoff, R. A. Hiatt, C. Gerdts. 2015. “Reducing under-reporting of stigmatized health events using the List Experiment: results from a randomized, population-based study of abortion in Liberia.” *International Journal of Epidemiology* 44 (6): 1951–1958. https://academic.oup.com/ije/article/44/6/1951/2572557.

^26^ Guttmacher. 2017. *Abortion in Asia.* New York, New York, USA: Guttmacher. https://www.guttmacher.org/sites/default/files/factsheet/ib_aww-asia_0.pdf

^27^ The American College of Obstetricians and Gynecologists. 2015. *Early Pregnancy Loss.* Washington, DC, USA: American College of Obstetricians and Gynecologists. https://www.acog.org/clinical/clinical-guidance/practice-bulletin/articles/2018/11/early-pregnancy-loss.

^28^ Assaf, S., M. Khawaja, J. Dejong, Z. Mahfoud, K. Yunis. 2009. “Consanguinity and reproductive wastage in the Palestinian Territories.” *Paediatric and Perinatal Epidemiology* 23 (2). https://doi.org/10.1111/j.1365-3016.2008.00988.x.

^29^ Gowri, V., A. M. Udayakumar, W. Bsiso, Y. Al Farsi, K. Rao. 2011. “Recurrent early pregnancy loss and consanguinity in Omani couples.” *Acta Obstetricia et Gynecologica Scandinavica* 90 (10): 1167–1169. https://doi.org/10.1111/j.1600-0412.2011.01200.x.

^30^ Al Husain, M. & M. Al Bunyan. 1996. “Consanguineous marriages in a Saudi population and the effect of inbreeding on prenatal and postnatal mortality.” *Annals of Tropical Paediatrics: International Child Health* 17 (2): 155–160. https://doi.org/10.1080/02724936.1997.11747879.

^31^ Saad, F. A. & E. Jauniaux. 2002. “Recurrent early pregnancy loss and consanguinity.” *Reproductive BioMedicine Online* 5 (2): 167–170. https://doi.org/10.1016/S1472-6483(10)61620-3. 

^32^ Ross, J. & Stover, J. 2013. “Use of modern contraception increases when more methods become available: analysis of evidence from 1982–2009.” *Global Health: Science and Practice* 1 (2): 203-2012. https://doi.org/10.9745/GHSP-D-13-00010.

^33^ Bruce, J. 1990. “Fundamentals of quality of care: a simple framework.” *Studies in Family Planning* 21(2): 61–90. https://doi.org/10.2307/1966669.

