# Supplementary material for: Shifting dynamics: Changes in the relationship between total fertility rate and contraceptive prevalence rate in Jordan between 2012 and 2017
Source: Gates Open Res. 2021 Jun 14;4:160. Originally published 2020 Oct 15. [Version 2] doi: 10.12688/gatesopenres.13188.2 (PMC8282988; doi:10.12688/gatesopenres.13188.2)
Supplement: Peer review history

# Peer review: Reviewer response for version 2

**DOI:** 10.21956/gatesopenres.14551.r30745

**Published:** 2021-07-19

**Authors:** Sara Riese

**Affiliations:** 1 ICF International, Fairfax, VA, USA

Thank you to the authors for considering my comments and modifying the article. With these additions, I believe that this paper should be accepted for indexing. Although it does not identify a clear answer to the overall research question, it does explore and rule out many of the potential explanations. In this way, it is a contribution to the literature to provide a framework for analysis for unexpected and unexplained patterns in survey results. Both the specific effort to understand these issues in the Jordan survey, as well as the framework for analysis, are valuable and worthy of indexing.

Is the work clearly and accurately presented and does it cite the current literature?

Yes

If applicable, is the statistical analysis and its interpretation appropriate?

Partly

Are all the source data underlying the results available to ensure full reproducibility?

Partly

Is the study design appropriate and is the work technically sound?

Partly

Are the conclusions drawn adequately supported by the results?

Yes

Are sufficient details of methods and analysis provided to allow replication by others?

Yes

Reviewer Expertise:

Implementation science, quality of care, reproductive health, equity

I confirm that I have read this submission and believe that I have an appropriate level of expertise to confirm that it is of an acceptable scientific standard.

# Peer review: Reviewer response for version 2

**DOI:** 10.21956/gatesopenres.14551.r30767

**Published:** 2021-07-15

**Authors:** Lindsay Mallick

**Affiliations:** 1 University of Maryland School of Public Health, College Park, MD, USA

Thank you for the opportunity to review this manuscript. This manuscript provides an incredibly comprehensive examination of factors that may explain the fertility related paradox in Jordan: that the total fertility rate has declined while contraceptive use has also declined in tandem. Although no one clear answer emerged as a result of this analysis, several important conclusions can still be drawn from analysis, for example, that these trends are not the results of major data quality issues. As researchers and stakeholders continue to explore and monitor these trends, this paper serves as an important contribution to the literature and guide for further research. As this paper has already undergone several revisions, I only have minor comments that could be addressed to improve the quality overall.

 The more substantial suggestions I have are:

- The various rates examined in the study are not well-defined. I suggest adding a text box or table, even as appendix, to define and describe the calculation of these rates.
- There were many different explanatory factors examined; however, not all were justified, described, or explained. Especially for exploring influencing factors on TFR/CPR section, as well as for examining ASFR, it would be helpful to connect each of these avenues of exploration to a specific hypothesis. Ordinarily this would be appropriate for the introduction, but as there were so many aspects studied in this analysis, some context in the methods section would be helpful. E.g., you do this for proximate determinants, but not population distribution or changes in fertility intentions.
- Although there do seem to be some explanations and the resulting estimates seem to be at least partially explained (EC under reported, marriage distribution change, marital separation, infecundity, possibly abortion). Is it not possible that the confluence of multiple, partial factors have contributed and together results in these more substantial trends?
    
- You note on p. 11 “the means of reducing unwanted fertility remains unexplained”; however, your analysis does uncover some potential explanations. In the results and discussion, you review these factors separately, but I think it would be important to note that there may not be one sole factor that contributes to this situation in Jordan; rather, a combination of many factors. Further, one potential factor that was not discussed—even as a data limitation or area for future research—is teasing out the contribution and role of male infertility. How might that play a role in your analysis and findings?
- I have some issues with the conclusion and recommendations. First, promoting an increase in contraceptive use and improved quality of care are not relevant to this paper. I believe the first two paragraphs of the conclusion are enough and the rest could be cut. However, if they are kept, I suggest connecting the recommendations listed more strongly with meeting women’s fertility desires; although I don’t believe this was the intent of the authors, this conclusion could otherwise be read as promoting family planning for population control.

Minor comments:

- In the background section you mention service statistics but not CRVS and census data, which were also used.
- In the background section, you mention the impetus for the investigation is to direct future investments in family planning; however, this manuscript goes beyond that and helps to improve our understanding of population dynamics.
- For external validation using census data, how complete is birth and population data? In some countries, civil registrations and vital statistics systems are not fully functioning and don’t capture records for the total population.
- In the methods section (under external validation), consider describing FPET, maybe with a bit more detail, before you discuss how you input EMU. It’s important to know that survey data feed into FPET, to give context to why you left out 2017 (described in results but should be moved to methods). Also, spell out UMass.
- I suggest adding notes on figures to spell out acronyms for readers who are unfamiliar; Figure 6 should include what the pinkish purple and lavender purple bars are.
- For figure 6, the black dots are service statistics - is that EMU calculated from service statistics? Is the most recent point reflecting EMU is close to 20%? That may be helpful to describe.
- Define TAR acronym.
- When describing results examining method mix, first describe the current method mix overall (what are the dominant method(s) before describing changes.)
- The DHS Program is no longer MEASURE DHS, note new web address. Also, the ‘t’ in the DHS Program is also capitalized (in 2 ^nd^ paragraph of methods).

Is the work clearly and accurately presented and does it cite the current literature?

Yes

If applicable, is the statistical analysis and its interpretation appropriate?

Yes

Are all the source data underlying the results available to ensure full reproducibility?

Partly

Is the study design appropriate and is the work technically sound?

Yes

Are the conclusions drawn adequately supported by the results?

Partly

Are sufficient details of methods and analysis provided to allow replication by others?

Yes

Reviewer Expertise:

Reproductive, maternal, and newborn health; quality of care, specifically quality of care for family planning

I confirm that I have read this submission and believe that I have an appropriate level of expertise to confirm that it is of an acceptable scientific standard.

# Peer review: Reviewer response for version 2

**DOI:** 10.21956/gatesopenres.14551.r30730

**Published:** 2021-06-28

**Authors:** Thomas W. Pullum

**Affiliations:** 1 Demographic and Health Surveys, ICF, Rockville, MD, USA

The authors have applied a wide range of strategies to explain the unexpected pattern of decline in CPR and decline in TFR between the two most recent DHS surveys in Jordan. They have successfully eliminated some potential explanations and have identified many other potential explanations that cannot be tested with the available data. The fact that the authors have not been able to account for the unexpected pattern does not invalidate the effort.

 Since the release of the 2017-18 survey, the staff of DHS have independently applied many of the approaches used by Bietsch *et al.* We have been particularly concerned with possible incompatibility of the sampling frames for the two surveys, possible irregularities during fieldwork, and other evidence of data quality issues. We have been unable to identify any serious problems. However, the search continues. If there are problems with the data, we definitely do not want them to recur in the next survey.

 If the unexpected pattern does not arise from data quality flaws, then it must be due to the kinds of factors Bietsch *et al.* explore, such as compositional change between the surveys, the asynchronicity of recent fertility and current use, changes in the method mix or use-effectiveness, a systematic pattern of under-reporting of contraceptive use or of other pregnancy terminations, etc. Thus far, none of those potential explanations appears to be sufficient.

 Eventually—at the latest, after the next survey—there will be a reconciliation of the inter-related trajectories of fertility intentions, contraceptive use, and fertility in Jordan, overall and in sub-populations. Until then, it is important to have a published record of the efforts to understand the puzzle, even if they are not completely successful. 

 Page 2, column 2, line 4: “One DHS further analysis on fertility in Nepal.” This fragment needs to be fixed. I will not refer to other editing issues, but there are several.

 For more than ten years, the URL www.measuredhs.com has been automatically redirected to www.dhsprogram.com. MEASURE DHS is an obsolete label for The DHS Program.

Is the work clearly and accurately presented and does it cite the current literature?

Yes

If applicable, is the statistical analysis and its interpretation appropriate?

Yes

Are all the source data underlying the results available to ensure full reproducibility?

Yes

Is the study design appropriate and is the work technically sound?

Yes

Are the conclusions drawn adequately supported by the results?

Yes

Are sufficient details of methods and analysis provided to allow replication by others?

Yes

Reviewer Expertise:

Demographic methods, analysis of demographic survey data

I confirm that I have read this submission and believe that I have an appropriate level of expertise to confirm that it is of an acceptable scientific standard.

# Peer review: Reviewer response for version 2

**DOI:** 10.21956/gatesopenres.14551.r30744

**Published:** 2021-06-16

**Authors:** Issa Masarweh

**Affiliations:** 1 Arab Institute for Training and Research in Statistics, Amman, Jordan

After reviewing the new version of the research “Shifting dynamics: Changes in the relationship between total fertility rate and contraceptive prevalence rate in Jordan”, I believe that the authors have not addressed the many concerns/comments I raised earlier.

 The authors challenged a well-established and strong association between TFR and CPR but could not provide sound and convincing evidences on their thesis of “Shifting Dynamics” in this association.

 Thus, I reaffirm the status of “Not Approved”.

Is the work clearly and accurately presented and does it cite the current literature?

Yes

If applicable, is the statistical analysis and its interpretation appropriate?

No

Are all the source data underlying the results available to ensure full reproducibility?

Partly

Is the study design appropriate and is the work technically sound?

Partly

Are the conclusions drawn adequately supported by the results?

No

Are sufficient details of methods and analysis provided to allow replication by others?

Partly

Reviewer Expertise:

NA

I confirm that I have read this submission and believe that I have an appropriate level of expertise to state that I do not consider it to be of an acceptable scientific standard, for reasons outlined above.

# Peer review: Reviewer response for version 1

**DOI:** 10.21956/gatesopenres.14393.r29824

**Published:** 2020-11-10

**Authors:** Sara Riese

**Affiliations:** 1 ICF International, Fairfax, VA, USA

The article offers an analysis that attempts to understand and identify the inconsistency in the declining TFR alongside a declining CPR in the most recent Jordan PFHS. The paper looks at this question in two ways: first by attempting to validate the TFR and CPR data from the Jordan PFHS, and second by exploring factors traditionally associated with TFR and CPR, including Bongaarts’ proximate determinants of fertility.

 In the data validation section, while the Schoumaker 2014 Methodological Report is cited and possible issues around birth history collection are mentioned, there are a number of other possible data quality issues highlighted in from that report which would have been good to mention in this paper, for example sample design and quality of age reporting. While I don’t necessarily know if these would clearly identify issues that would clarify the TFR/CPR trends in this case, when conducting data quality checks, they would have been informative to mention and/or explore. A more thorough discussion of these issues would be valuable, especially given the lack of any other significant explanation.

 In addition, when presenting point estimates for TFR, it would be appropriate to include the CIs.

 Finally, in the introduction section, the authors state that Jordan is not the only country where there are shifts to the typical relationship between TFR and CPR. It would have been helpful to have some discussion of other examples of this situation and whether or not explanations have been identified in those cases.

 In general, the study is a clear contribution to the literature as it attempts to systematically approach this counter-intuitive finding of decreasing TFR and CPR. The authors have done well in their presentation of the results and discussion of the inconclusive findings. My “partly” responses on the study design and statistical analysis questions reflect that I believe this study would benefit from a more thorough discussion or exploration of the potential data quality issues around TFR estimates, as well as a discussion of other country cases. The “partly” response on the underlying source data is a reflection of the fact that the Jordanian census data is not openly available to the public.

Is the work clearly and accurately presented and does it cite the current literature?

Yes

If applicable, is the statistical analysis and its interpretation appropriate?

Partly

Are all the source data underlying the results available to ensure full reproducibility?

Partly

Is the study design appropriate and is the work technically sound?

Partly

Are the conclusions drawn adequately supported by the results?

Yes

Are sufficient details of methods and analysis provided to allow replication by others?

Yes

Reviewer Expertise:

Implementation science, quality of care, reproductive health, equity

I confirm that I have read this submission and believe that I have an appropriate level of expertise to confirm that it is of an acceptable scientific standard, however I have significant reservations, as outlined above.

# Response

**Published:** 2021-06-07

**Authors:** Kristin Bietsch

The authors are very grateful for Dr. Riese's careful and constructive comments. Our responses are as follows:

*We have included additional examples of data quality issues that may contribute to an underestimation of TFR, and feel that our analysis constructing TFRs from the last three surveys assesses the presence of these data quality concerns. If any of the data quality issues mentioned in Schoumaker 2014 were present in any of the surveys, we would expect the TFR estimates from that survey to appear as outliers, but our analysis shows that the TFRs calculated from each survey are consistent.*

*We have included CIs for all the tables and figures that present TFR (Table 1, Figure 1, Figure 2, Table 3 and Table 4).*

*We expanded this section of the introduction to include a list of countries where there were shifts in the typical relationship between TFR and CPR (which we identified using STATcompiler), and did a literature search to try to identify whether explanations have been identified in these cases. We identified one study of fertility in Nepal.*

*We thank Dr. Riese for reviewing our paper and for her valuable feedback. We hope the changes we made to the paper have sufficiently addressed her concerns.*

# Peer review: Reviewer response for version 1

**DOI:** 10.21956/gatesopenres.14393.r29827

**Published:** 2020-10-29

**Authors:** Issa Masarweh

**Affiliations:** 1 Arab Institute for Training and Research in Statistics, Amman, Jordan

Comments on my answer to Question 4: The statistical methods used were inappropriate and the authors resorted to data analysis outside the survey to show that Jordan has experienced a shift in the typical relationship between CPR and TFR rather than data errors.

**Early Questioning of 2017/18 DHS Data?**

 Long before releasing JPFHS 2017/18 (DHS) main report, Jordan Department of Statistics (the survey owner) organized a meeting attended by a large number of stakeholders representing GOs, NGOs, academicians and donor agencies working in Jordan to discuss the prelim results of the survey. Many of the attendees have then raised questions about the survey TFR result (2.6 for the Jordanian sample and 2.7 for the national sample) and believed it to be inconsistent with all direct or intermediate variables affecting fertility.

 The Department of Civil Status and Passport (in charge of Jordan vital register) published in its annual report of 2017 a TFR of 3.1 births per Jordanian woman (above JPFHS 2017/18 figure of 2.6) and a CBR of 24.2 births per 1000 compared to 21.6 (the survey figure).

**Post 2017/18 DHS of Release of Results:**

 TFR in two governorates of the North and South Region of Jordan is inconsistent with their mCPR and unmet need for FP. The formers have a high TFR (3.5 each) but the highest mCPR (42.5 and 41.1%) and the lowest unmet need (10.5 and 9.9 %) among all governorates of Jordan; while the South region has a TFR of 2.6 below the national TFR (2.7) but has the lowest mCPR (33.6%) among the three regions of Jordan (the lowest mCPR was 24.7% in the southern governorate of Ma'an).

**Multi-Method for Evaluating 2017 TFR Figure:**

 To verify the questionable 2017/2018 DHS TFR of 2.6 for Jordanian woman, I carried out an evaluation employing the following 8 methods (results are shown later in this report):

1. Cross-tab the 2012 and 2017/18 fertility variables against their correlates to assess the changes that took place; the inter-survey results were inconsistent.
2. Considering the 2015 Census fertility figure.
3. Calculating the proximate determinants of fertility indexes for 2012 and 2017/2018.
4. Estimating CPR and mCPR required for having a TFR of 2.6.
5. Running a regression of TFR against mCPR using the 7 DHSs 1990-2017 figures.
6. Recalculating 2017 TFR using the 2017/18 proximate determinants of fertility figures.
7. Estimating 2017 TFR from data on births by age of mother from recent Jordan vital statistics record.
8. Calculating CYP from Jordan FP Contraceptive Logistics System data for the years 2012-2018.

**Below are the changes in the direct factors affecting TFR between 2012 and 2017/18 Survey which indicate that TFR should have been rising rather than declining:**

- % of ever-married WRA increased by 2 percentage points.
- % of currently married WRA increased by 1.5 percentage points.
- % of currently WRA who are sexually active increased by 3 percentage points.
- % currently pregnant increased from 5.5% to 5.9%;
- % started childbearing at age 15-17 increased from 2.9% to 3.1%.
- mCPR decreased by 4 percentage points;
- Unmet need for using contraceptives increased by 2 percentage points;
- Median age at F.S increased by 0.3 years;
- % of non-user of FP methods among currently married WRA who don’t intend to use contraceptives increased by 15 percentage points;
- % of ever-married WRA who didn’t discuss FP with health professionals in the last 12 months increased by 4 percentage points;
- Childless women 45-49 years (proxy of sterility) decreased by 2 percentage points;
- C-S deliveries decreased by 1.4 percentage points (means shorter pregnancy interval);
- Couple years of protection CYP (proxy of modern contraceptives users) based on Jordan MoH FP Logistic System decreased from 229,000 to 224,000 between 2012 and 2017.

**Estimated TFR for 2017 based on:**

1. Bongaarts five proximate determinants of fertility - TFR is 3.24 births per women (not 2.6);
2. Direct estimate using published data for 2017 by the Department of Civil Status and Passport yielded a figure of 3.56 birth per woman (not 2.6);
3. TFR figure from 2015 Census data is 3.4 (3.38) births per woman and it is impossible for TFR to decline to 2.6 or 2.7 in two years;
4. The empirical relationship between TFR and the proximate determinants of fertility; in order to have a TFR of 2.6, mCPR should have been 46% in 2017 and not 38% as 2017 JPFH has shown;
5. Two indirect methods for estimating TFR for 2017, I got the following results:

- Rele method – 3.15 births per women.
- Using the survey reported ASFRs, number of WRA, mid-year total population and number of Jordanian annual births, TFR was 3.56 births per woman (same as the figure derived from the direct method).

**One Possible Partial Explanation of the Rapid Fertility Decline 2012-2017 is the age structure of survey sample:**

 Jordan 2017 DHS interviewed a lower proportion of younger women and higher proportion of older Jordanian women of reproductive age compared with the 2012 DHS. This may partially explain the very low TFR resulted from later survey data.

 JPFHS 2017/2018 compared with 2012 JPFHS interviewed lower proportion of younger women (< 30 years) – 27.8 % vs. 30.7% who are more fecund and also want to have more children; and higher proportion of older women (35.1% vs. 32.7%) who are less fecund (40-49 years) and probably achieved their desired number of children.

**My Conclusions:**

 The authors’ arguments refute the well-established empirical findings in the literature regarding the inhibiting effect of contraceptives use on TFR; beside the impact of other proximate/direct variables. The findings presented in the article do not provide compelling evidence that the inter-survey decline in Jordan TFR is real and a result of shifting pattern.

 To avoid pregnancy, fecund women should refrain from sexual intercourse and/or use a contraceptive method. Accepting the authors’ inference that TFR may rapidly decline, the case of Jordan (other things being equal) without a decline in sexual exposure and significant increase in modern contraceptive use will give a false recommendation or approach to FP program managers and decision makers for not investing in FP services.

 Data errors in the later survey is not ruled out and it has been noticed by ICFI during the data collecting stage. Jordan fertility is still stalling; a trend witnessed in other countries.

Is the work clearly and accurately presented and does it cite the current literature?

Yes

If applicable, is the statistical analysis and its interpretation appropriate?

No

Are all the source data underlying the results available to ensure full reproducibility?

Partly

Is the study design appropriate and is the work technically sound?

Partly

Are the conclusions drawn adequately supported by the results?

No

Are sufficient details of methods and analysis provided to allow replication by others?

Partly

Reviewer Expertise:

Demographic analysis, analysis of fertility related data and formulation of population policy.

I confirm that I have read this submission and believe that I have an appropriate level of expertise to state that I do not consider it to be of an acceptable scientific standard, for reasons outlined above.

# Response

**Published:** 2021-06-07

**Authors:** Kristin Bietsch

The authors greatly respect Dr. Masarweh and are honored that he agreed to review the paper. We appreciate that he took the time to check other sources to prepare his review. We have acknowledged many of the trends Dr. Masarweh detailed in his review in our paper and agree that these trends should point to an increase in TFR. Interestingly, this is not what we have found in the JPFHS, which we validated internally and externally (using the 2015 census and annual birth numbers from the Jordanian Department of Statistics). The counter-intuitive trends between TFR and mCPR are what prompted this analysis. We stand by our analysis and have explored many of the proximate determinants of fertility as well as other potential changes to fertility with the DHS data.

 We agree that contraception is an important element of any family planning program and that maintaining the apparent low fertility will require continued strong efforts toward increased family planning use. In our conclusions and recommendations section we emphasize the importance of a continued focus on family planning programs, including improving quality, expanding the method mix and dispelling myths and misconceptions to help women achieve their fertility intentions.
